# Geographic pattern of the prevalence of intimate partner violence against women in Zanjan (Iran)

**DOI:** 10.3389/fpsyg.2024.1347077

**Published:** 2024-04-18

**Authors:** Farzaneh Karamitanha, Farzane Ahmadi, Vahid Fallah Abadi

**Affiliations:** ^1^Department of Community Medicine, Faculty of Medicine, Zanjan University of Medical Sciences, Zanjan, Iran; ^2^Department of Biostatistics and Epidemiology, Faculty of Medicine, Zanjan University of Medical Sciences, Zanjan, Iran; ^3^Department of Environmental Health Engineering, School of Public Health, Zanjan University of Medical Sciences, Zanjan, Iran

**Keywords:** intimate partner violence, physical violence, psychological violence, sexual violence, economic violence, geographic pattern

## Abstract

**Introduction:**

Intimate partner violence (IPV) against women is a serious public health issue and refers to physically, sexually and psychologically harmful behaviors as well as emotionally controlling behaviors and financial abuse that occur in the form of marriage or cohabitation. Knowing the current situation of the IPV prevalence against women and high-risk areas in the Zanjan city, Iran, can help policymakers to establish better health programs for risk reduction.

**Methods:**

This population-based cross-sectional study consisted of married women aged 18–55 years living in Zanjan city in 2021. 760 married women covered by 19 urban comprehensive health service centers (UCHSCs) were selected by the stratified systematic random sampling method. The prevalence of IPV against women was measured in four types: psychological, physical, sexual, and economic.

**Results:**

Mean (SD) age of the women was 35.49 (8.76) years. 606 women (79.7%) experienced one type of IPV. The highest and lowest IPV prevalence against women were psychological (76.6%) and economic (12%), respectively. The highest and lowest prevalence of psychological violence were observed in CUHSCs 2 and 17, physical violence in CUHSCs 1 and 14, sexual violence in CUHSCs 2 and 17, and economic violence in CUHSCs 2 and 8, respectively. The severity of violence was higher among self-employment or workers husbands, with low monthly household income, and among younger women.

**Discussion:**

The IPV rate in the target population is high, and the highest rate is related to psychological violence. These results highlight the need to intervention in the society and high-risk women for policymakers of the health system.

## Introduction

Intimate partner violence (IPV) against women is a serious public health issue and refers to physically, sexually and psychologically harmful behaviors as well as emotionally controlling behaviors and financial abuse occur in the form of marriage or cohabitation (Sardinha et al., 2022). Violence against women causes physical and psychological damage to women in the short and long term. It can cause physical injury, depression, anxiety, sexually transmitted infections, unwanted pregnancy and even death (Vos et al., 2015; Bacchus et al., 2018). The reported prevalence of this serious health problem varies between countries, likely due to the influence of cultural and social context. In reports from the United States, Canada, North American countries, Europe, and Southeast Asia, the physical violence prevalence is estimated to be between 16 and 46% (Davoudi et al., 2014). According to the 2018 CDC report, approximately 1 in 4 women and nearly 1 in 10 men have experienced domestic violence (DV), including sexual violence, physical violence, or stalking, in their lifetime (Smith et al., 2018). In Davoudi et al.’s study, DV in different cities of Iran was reported as 4.42–6.14% (Davoudi et al., 2014). In study conducted in Kerman, Iran, psychological violence had the highest rate of all types of violence (Gohari et al., 2023).

People of all races, cultures, genders, socio-economic classes and religions can experience IPV. Various underlying factors have been proposed to explain this public health and social problem. Economic instability, unsafe housing, neighborhood violence, lack of safe and stable child care, and lack of social support can make it worse. Economic independence is an important factor in preventing violence (Evans et al., 2020). Increasing age of the spouse, increasing the number of children, living in the village, poverty, divorce, having to rent a house and history of DV in the family of the spouses before marriage were also mentioned as relevant factors (Moazen et al., 2019; Sanz-Barbero et al., 2019; Chernet and Cherie, 2020).

Considering the magnitude of the problem and the importance of knowing the current situation regarding the IPV prevalence against women, this research aims to estimate the prevalence and identify high-risk areas of IPV in Zanjan city, Iran.

## Materials and methods

### Study design, participants and data collection

**MAP 1:**
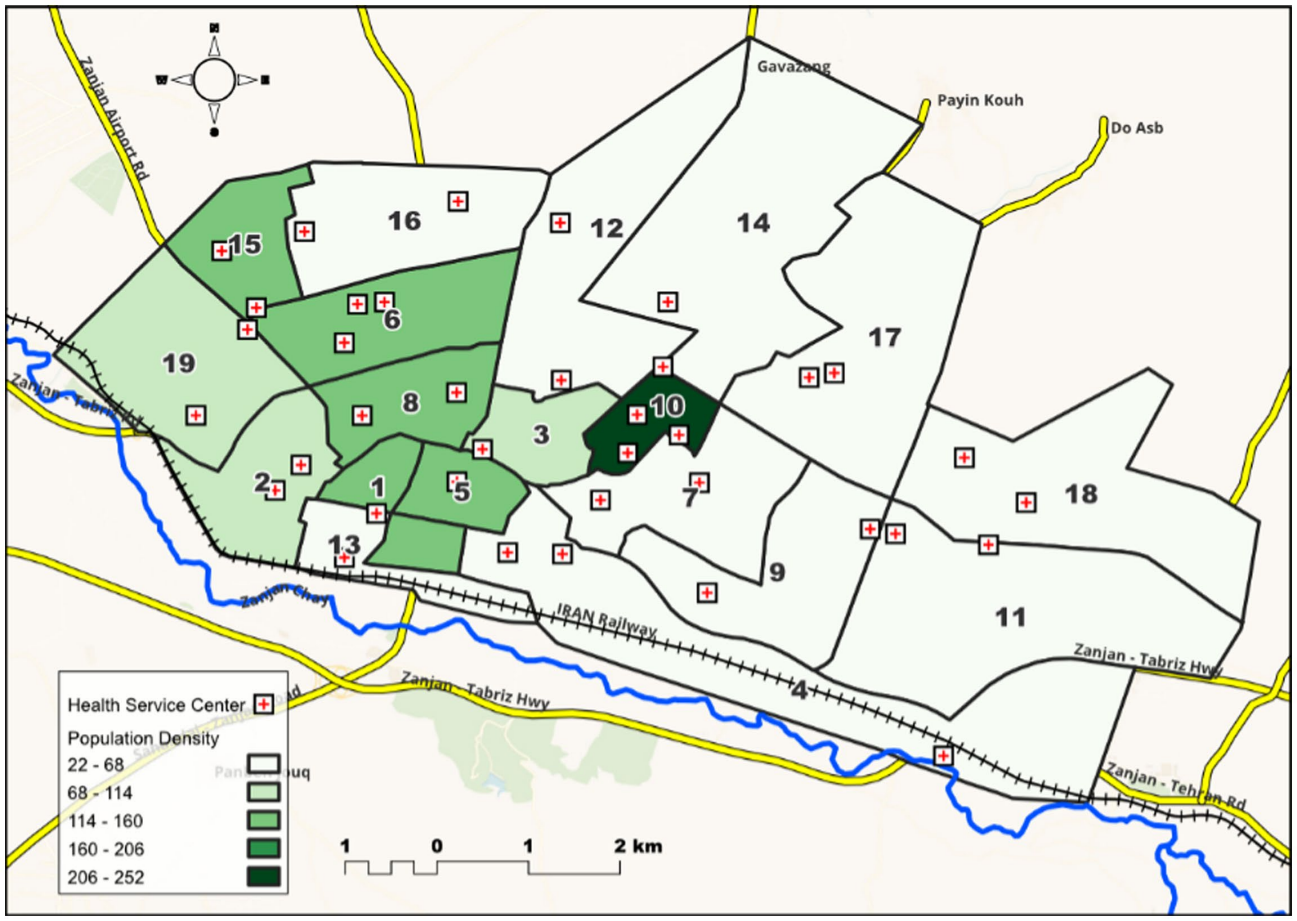
The population density of 19 comprehensive urban health service centers (population to the area covered by each center) (in terms of people per hectare).

This is a cross-sectional population-based study, studding a population of married women aged 18–55 years living in Zanjan city in 2021. Zanjan city is the capital of Zanjan province, one of the northwestern provinces of Iran and located west of Tehran (Iran’s capital). The study sample was selected from married women at 19 comprehensive urban health service centers (CUHSCs). According to the Iranian Statistics Center, the population of Zanjan city is 463,600 people in 2021. Map 1 shows the population density of each CUHSC (ratio of population to area of each CUHSC). In terms of population, CUHSC 18 has the largest population with 31,568 people and CUHSC 13 has the smallest population with 8,474 people. On average, each CUHSC covered 19,437 people (with a standard deviation of 7,581). In terms of size, CUHSC 4 has the largest size (675 hectares) and CUHSCs 5 and 10 has the smallest size (94 hectares). In addition, in terms of the population density per hectare, CUHSC 10 with a population density of 252 and CUHSC 4 and 12 with a population density of 22 have the lowest population density. To determine the population density and geometric characteristics of the investigated areas, Open Street Map was used in QGIS software. Also, in this software, WGS 84 / UTM zone 39 N image system was used, which is the most suitable imaging system for Zanjan city.

Based on the Cochran’s formula and with α = 0.05, *P* = 0.35—DV prevalence against women as reported by UN World Health Organization (2014)—, and d = 0.1P, the minimum sample size was estimated to be 715 women. A stratified systematic random sampling method was used, which CUHSCs were the strata. On average, in each CUHSC selected 40 married women, the sample size was 760 people (the number of samples for each CUHSC ranged from 37 to 43). After determining the number of people belonging to each CUHSC based on the formula $k_{i}=\frac{N_{i}}{n_{i}},\;i=1,\;\ldots ,\;19$ , where $k_{i}$ is the sampling interval, $N_{i}$ is the number of people belonging to a CUHSC, $n_{i}$ is the required sample size, the sampling interval is determined in each CUHSC. Based on this, the household number was extracted. If the married woman met the inclusion criteria, she was interviewed to complete the questionnaire.

Inclusion criteria were married women aged 18–55 years living in Zanjan city (at least in the last year) in 2021, living with their spouse for at least one year, and who agreed to participate study. The questionnaire was completed by an interviewer who has a master’s degree in clinical psychology and is familiar with interview methods. Data collection was performed using structured interviews in a private location. It took about 15 min to complete each questionnaire.

### Instrument

Data were collected using a demographic information form including women’s age, spouse’s and women’s occupation, spouse’s and women education level, spouse’s and women’s addiction, housing type (rented/owned) and monthly household income (million Toman) and violence against women questionnaire, that designed by Haj-Yahia (2000). The questionnaire was designed based on factors affecting the occurrence of violence and types of violence with 32 questions with 3 answer options: never, once, twice and more. It measures four types of violence in the past year, including psychological (items 1–16), physical (items 17–27), sexual (items 28–30), and economic (items 31–32).

IPV scores were analyzed quantitatively and qualitatively. To measure the prevalence of different types of IPV, scores for each item were dichotomous: 0 = never, 1 = at least once. Then, cumulative scores were calculated for each type of violence. Psychological violence was assessed according to four levels: not abused (wife had never experienced any of these acts), mild (wife had suffered from 1 to 5 acts); moderate (wife had to endure from 6 to 10 acts), and severe (wife had endure from 11 to 16 acts). Physical violence was assessed at three levels: not abused (wife has never been subjected to any of these acts), moderate (wife suffered from 1 to 6 acts), and severe (wife suffered from 7 to 11 acts). Sexual violence was assessed on three levels: not abused (wife has never been subjected to any of these acts), mild (the wife has been subjected to one act), and severe (wife has been subject to 2–3 acts). Economic violence was assessed at two levels as: not abused (wife has never been abused economically) and abused (the woman is abused by at least 1 act). Additionally, to compare IPV in different CUHSCs based on the number of repetitions of each abuse item, scores for each item were considered as 0 = never, 1 = once and twice and more = 2 (the higher the score, the more violence). Score of each type of IPV were calculated by summing the scores of related items. On this basis, the total violence score ranges from 0 to 64, psychological from 0 to 32, physical from 0 to 22, sexual from 0 to 6, and economic from 0 to 4. This questionnaire was validated and relabeled in the Iranian population by Khosravi and KhaghaniFard (Khosravi and Khaghani Fard, 2004). Cronbach’s alpha ranged from 0.71 to 0.93 for the four types of violence (Khojasteh Mehr et al., 2021). In this study, Cronbach’s alphas were 0.84, 0.80, 0.81, 0.60, and 0.90 for psychological, physical, sexual, economic and total of violence, respectively.

### Statistical analysis

The normality of IPV in each CUHSC was assessed by Shapiro–Wilk test that indicated they had not normal distribution. The Kruskal-Wallis test was used to compare IPV in 19 CUHSCs. The pairwise Mann–Whitney post-hoc tests with Bonferroni correction are then used to find out which pairs of 19 CUHSCs were different. The similar CUHSCs in terms of IPV were placed in one group (subgroup). In fact, CUHSCs that did not have significant differences in terms of IPV score were considered together. In the end, the homogenous subgroups of 19 CUHSCs were determined. After that, the demographic and income variables compared based on the homogenous subgroups resulted from the total score of IPV by Chi-square and Kruskal-Wallis tests.

TwoStep cluster analysis was used to determine the socio-economic status of participates based on spouse’s education level and job status, husband’s addiction status, home ownership status, and monthly income level. Bayesian Information Criteria (BIC) was used to specify socio-economic level. The number of levels was selected that achieved the lowest BIC. The goodness of fit of the TwoStep cluster analysis was assessed using Silhouette measure of cohesion and separation, which is fair if it is greater than 0.2. This cluster analysis calculates the contribution of each variable to the identification and grouping individuals, which is called the predictor importance, and it ranges from 0 to 1. The higher the importance value, the more important the variable. After determining participates’ socio-economic status, we used the binary logistic regression to find UCHSCs that were homogenous with respect to socio-economic status. Here, the socio-economic status was the dependent variable and UCHSCs was the independent variable. The analysis was performed by SPSS 24. Also, the maps were prepared by the QGIS 3.16.5-Hannover software.

## Results

The mean (Standard Deviation, SD) age of the 760 women was 35.49 (8.76) years. The monthly household income of more than half of the participants was 5–10 million Tomans. About 82% of them were housewives and 62% of their husband were self-employed. Only one woman (0.1%) had addict, while this was 1.6% in their husbands. More than half of women and their husband had Diploma or less (70.1 and 68.6%, respectively), and 39.1% had rented housing (Table 1).

**Table 1:** Demographic and social variables of women and their husbands.

Variable	Category	Frequency (%)
**Women**		
Education level	Illiterate	56 (7.4)
	Under diploma	229 (30.1)
	Diploma	248 (32.6)
	Associate degree	178 (23.4)
	Bachelor	47 (6.2)
	Doctor	2 (0.3)
Job status	Housewife	621 (81.7)
	Employed	139 (18.3)
Addiction status	Yes	1 (0.1)
	No	759 (99.9)
**Husband**		
Education level	Illiterate	57 (7.5)
	Under diploma	230 (30.3)
	Diploma	234 (30.8)
	Associate degree	170 (22.4)
	Bachelor	53 (7.0)
	Doctor	16 (2.1)
Job status	Unemployed	12 (1.6)
	Government job	234 (30.8)
	Self-employed	473 (62.2)
	Daily worker	41 (5.4)
Addiction status	Yes	12 (1.6)
	No	748 (98.4)
**Household**		
Housing ownership status	Rental	297 (39.1)
	Owner	463 (60.9)
Monthly income (million Tomans)	< 5	180 (23.7)
	5–10	475 (62.5)
	10–15	50 (6.6)
	> 15	55 (7.2)

Based on the TwoStep cluster analysis, there were two levels of participates socio-economic (BIC = 7791.71 and Silhouette measure of cohesion and separation = 0.25). The education level of women and husbands, monthly income, and job status of women were important variables to determine socio-economic of women (predictor importance: 1.00, 0.89, 0.55, and 0.47, respectively). By comparing two groups of women’ socio-economic status over 19 UCHSCs using binary logistic regression, it identified that UCHSCs can be summarized into three homogenous subgroups (Table 2).

**Table 2:** Frequency (%) of women by homogenous subgroups of the CUHSCs socio-economic status of Zanjan city.

Homogeneous subgroups of socio-economic status of CUHSCs	UCHSC	Socio-economic status of participates	
		Low (*n* = 578)	High (*n* = 182)
1: Low	1	35 (87.5)	5 (12.5)
	2	37 (92.5)	3 (7.5)
	5	34 (82.9)	7 (17.1)
	6	37 (92.5)	3 (7.5)
	10	36 (92.3)	3 (7.7)
	13	36 (90.0)	4 (10.0)
	15	36 (90.0)	4 (10.0)
	16	33 (80.5)	8 (19.5)
	19	25 (61.0)	16 (39.0)
2: Medium	3	24 (64.9)	13 (35.1)
	4	33 (76.7)	10 (23.3)
	7	29 (70.7)	12 (29.3)
	8	31 (77.5)	9 (22.5)
	9	28 (68.3)	13 (31.7)
	11	30 (73.2)	11 (26.8)
	12	20 (54.1)	17 (45.9)
	17	25 (61.0)	16 (39.0)
	18	28 (70.0)	12 (30.0)
3: High	14	8 (20.5)	31 (79.5)

CUHSCs, comprehensive urban health service centers.

In total, 606 women (79.7%) experience one type of IPV such that 48.2% experienced one type of IPV, 18.8% experienced two types of IPV, 7.5% experienced three types of IPV and 5.3% experienced four types of IPV. The highest and lowest IPV prevalence against women were psychological with 76.6% and economic with 12%, respectively. The prevalence of different types of IPV across CUHSCs is reported in Table 3. The highest and lowest prevalence of psychological violence was observed in CUHSCs 2 and 17, physical violence in CUHSCs 1 and 14, sexual violence in CUHSCs 2 and 17, and economic violence in CUHSCs 2 and 8, respectively.

**Table 3:** Prevalence of IPV against women by 19 CUHSCs of Zanjan city.

CUHSC	Violence											
	Psychological				Physical			Sexual			Economic	
	Not abused	Mild	Moderate	Sever	Not abused	Moderate	Sever	Not abused	Mild	Sever	Not abused	Abused
1	3 (7.5)	22 (55.0)	13 (32.5)	2 (5.0)	17 (42.5)	21 (52.5)	2 (5.0)	24 (60.0)	4 (10.0)	12 (30.0)	28 (70.0)	12(30.0)
2	0 (0.0)	3 (90.0)	1 (2.5)	3 (7.5)	28 (70.0)	9 (22.5)	3 (7.5)	17 (42.5)	8 (20.0)	15 (37.5)	25 (62.5)	15(37.5)
3	7 (18.9)	26 (70.3)	3 (8.1)	1 (2.7)	27 (73.0)	7 (18.9)	3 (8.1)	32 (86.5)	2 (5.4)	3 (8.1)	33 (89.2)	4 (10.8)
4	12 (27.9)	25 (48.1)	3 (7.0)	3 (7.0)	38 (88.4)	2 (4.7)	3 (7.0)	37 (86.0)	2 (4.7)	4 (9.3)	39 (90.7)	4 (9.3)
5	10 (24.4)	24 (58.5)	5 (12.2)	2 (4.9)	30 (73.2)	9 (22.0)	2 (4.9)	37 (90.2)	0 (0.0)	4 (9.8)	37 (90.2)	4 (9.8)
6	9 (22.5)	26 (65.0)	4 (10.0)	1 (2.5)	32 (80.0)	7 (17.5)	1 (2.5)	28 (70.0)	1 (2.5)	11 (27.5)	37 (92.5)	3 (7.5)
7	15 (36.6)	18 (43.9)	2 (4.9)	6 (14.6)	32 (78.0)	4 (9.8)	5 (12.2)	34 (82.9)	0 (0.0)	7 (17.1)	34 (82.9)	7 (17.1)
8	7 (17.5)	31 (77.5)	1 (2.5)	1 (2.5)	36 (90.0)	4 (10.0)	0 (0.0)	35 (87.5)	3 (7.5)	2 (5.0)	40 (100.0)	0 (0.0)
9	10 (24.4)	29 (70.7)	1 (2.4)	1 (2.4)	40 (97.6)	1 (2.4)	0 (0.0)	35 (85.4)	4 (9.8)	2 (4.9)	40 (97.6)	1 (2.4)
10	13 (33.3)	23 (59.0)	0 (0.0)	3 (7.7)	35 (89.7)	1 (2.6)	3 (7.7)	34 (87.2)	3 (7.7)	2 (5.1)	35 (89.7)	4 (10.3)
11	7 (17.1)	28 (68.3)	4 (9.8)	2 (4.9)	34 (82.9)	4 (9.8)	3 (7.3)	33 (80.5)	2 (4.9)	6 (14.6)	36 (87.8)	5 (12.2)
12	14 (37.8)	20 (54.1)	0 (0.0)	3 (8.1)	34 (91.9)	2 (5.4)	1 (2.7)	34 (91.9)	1 (2.7)	2 (5.4)	35 (94.6)	2 (5.4)
13	9 (22.5)	25 (62.5)	5 (12.5)	1 (2.5)	35 (87.5)	5 (12.5)	0 (0.0)	34 (85.0)	4 (10.0)	2 (5.0)	36 (90.0)	4 (10.0)
14	10 (25.6)	27 (69.2)	1 (2.6)	1 (2.6)	36 (92.3)	1 (2.6)	2 (5.1)	35 (89.7)	1 (2.6)	3 (7.7)	37 (94.9)	2 (5.1)
15	6 (15.0)	28 (70.0)	3 (7.5)	3 (7.5)	34 (85.0)	6 (15.0)	0 (0.0)	24 (60.0)	5 (12.5)	11 (27.5)	34 (85.0)	6 (15.0)
16	8 (19.5)	27 (65.9)	5 (12.2)	1 (2.4)	32 (78.0)	7 (17.1)	2 (4.9)	24 (58.5)	4 (9.8)	13 (31.7)	38 (92.7)	3 (7.3)
17	17 (41.5)	20 (48.8)	4 (9.8)	0 (0.0)	36 (87.8)	4 (9.8)	1 (2.4)	41 (100.0)	0 (0.0)	0 (0.0)	40 (97.6)	1 (2.4)
18	10 (25.0)	23 (57.5)	7 (17.5)	0 (0.0)	30 (75.0)	10 (25.0)	0 (0.0)	35 (87.5)	2 (5.0)	3 (7.5)	39 (97.5)	1 (2.5)
19	11 (28.2)	22 (56.4)	5 (12.8)	1 (2.6)	32 (82.1)	6 (15.4)	1 (2.6)	19 (48.7)	12 (30.8)	8 (20.5)	26 (66.7)	13 (33.3)
Total	178 (23.4)	480(63.2)	67 (8.8)	35(4.6)	618 (81.3)	110 (14.5)	32(4.2)	592 (77.9)	58 (7.6)	110(14.5)	669 (88.0)	91 (12.0)

IPV, intimate partner violence; CUHSCs, comprehensive urban health service centers.

**MAP 2:**
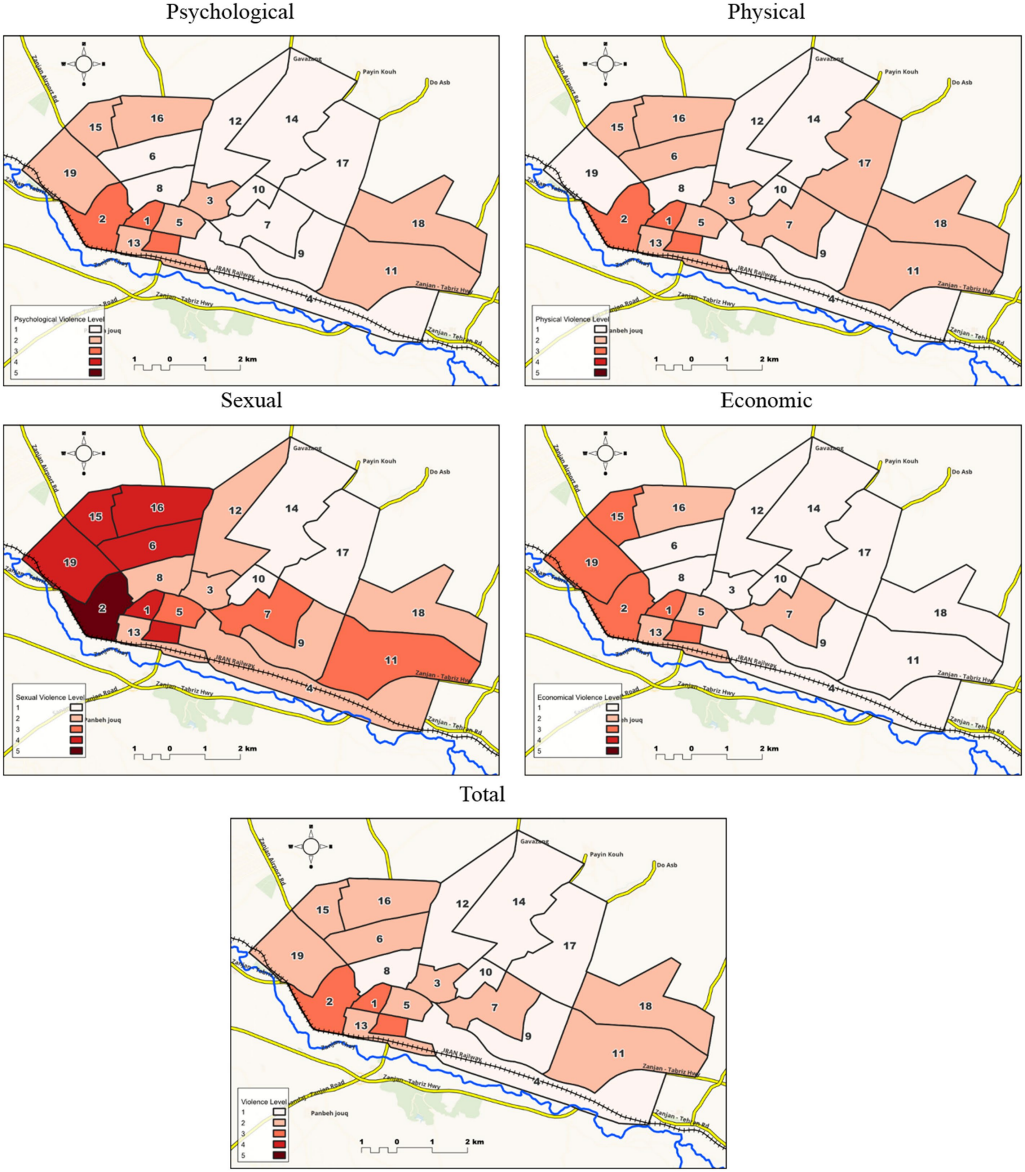
Homogeneous subgroups obtained from 19 comprehensive urban health service centers (CUHSC) in Zanjan city in terms of intimate partner violence against women (1: the lowest and 3 or 5: the highest value of violence).

To compare IPV against women in different CUHSCs, mean (SD) of violence by CUHSCs is shown in Table 4. In total, the mean (SD) of violence was 6.43 (8.74), ranged from 0 to 63. CUHSCs 1 and 2 had the highest and CUHSC 14 had the minimum of violence. Among 19 CUHSCs, there were significant differences in the four types and total score of violence (*p* < 0.001). Additionally, 19 CUHSCs were compared pairwise using the Mann–Whitney test with Bonferroni correction to determine homogenous subgroups of CUHSCs. Based on the results of the post-hoc test, CUHSCs were classified and their homogenous subgroups were determined. Violence scores had significant differences between subgroups. The mean (SD) and number of participants in each homogenous subgroups were reported in Table 5. The number of subgroups variable from 3 to 5 by violence components. The first and the last subgroup had the minimum and maximum level of violence, respectively. The name of CUHSCs in each homogenous subgroups were depicted in Map 2.

**Table 4:** Mean (SD) of IPV against women by 19 CUHSCs of Zanjan city.

CUHSCs	Violence				
	Psychological	Physical	Sexual	Economic	Total
1	8.17 (6.26)	2.25 (3.49)	1.37 (1.94)	0.55 (0.99)	12.35 (9.75)
2	7.17 (5.65)	2.10 (5.02)	2.20 (2.3)	0.87 (1.32)	12.35 (11.89)
3	4.54 (4.27)	1.38 (3.61)	0.30 (1.17)	0.19 (0.74)	6.40 (8.68)
4	3.84 (5.59)	0.56 (3.35)	0.21 (0.77)	0.16 (0.75)	4.77 (9.56)
5	5.37 (6.54)	1.90 (5.06)	0.51 (1.63)	0.27 (0.92)	8.05 (12.31)
6	4.40 (3.58)	0.97 (2.35)	1.38 (2.32)	0.10 (0.44)	6.85 (6.01)
7	4.44 (7.61)	1.00 (3.73)	0.54 (1.67)	0.24 (0.89)	6.22 (11.93)
8	2.82 (2.92)	0.30 (1.16)	0.35 (1.00)	0.00 (0.00)	3.47 (3.93)
9	2.56 (2.73)	0.15 (0.94)	0.29 (0.87)	0.02 (0.16)	3.02 (3.47)
10	2.74 (4.15)	0.61 (3.53)	0.15 (0.54)	0.08 (0.35)	3.59 (6.60)
11	5.00 (5.34)	0.85 (3.09)	0.61 (1.66)	0.15 (0.53)	6.61 (8.04)
12	2.59 (4.16)	0.08 (0.36)	0.22 (1.03)	0.03 (0.16)	2.92 (5.38)
13	4.55 (5.14)	0.85 (2.75)	0.37 (1.00)	0.25 (0.81)	6.03 (7.66)
14	2.54 (2.85)	0.20 (1.28)	0.13 (0.57)	0.00 (0.00)	2.87 (3.89)
15	6.00 (6.40)	0.92 (2.71)	1.65 (2.30)	0.35 (0.89)	8.92 (10.50)
16	5.15 (5.77)	1.56 (3.89)	1.71 (2.26)	0.24 (0.92)	8.66 (9.66)
17	3.41 (4.24)	0.90 (2.95)	0.00 (0.00)	0.05 (0.31)	4.37 (6.55)
18	5.75 (5.50)	0.95 (2.11)	0.40 (1.37)	0.05 (0.32)	7.15 (7.23)
19	4.54 (4.68)	0.72 (2.13)	1.33 (1.59)	0.77 (1.24)	7.36 (7.63)
Test statistic	81.94	79.85	135.01	94.34	117.56
*p*-value^a^	< 0.001	< 0.001	< 0.001	< 0.001	< 0.001

a Kruskal-Wallis test.

SD, standard deviation; IPV, intimate partner violence; CUHSC, comprehensive urban health service centers.

**Table 5:** Mean (SD) and number of women (*n*) of IPV against women by homogenous subgroups of the CUHSCs of Zanjan city.

Violence	Total	Homogeneous Subgroups									
		1		2		3		4		5	
	Mean (SD)	*n*	Mean (SD)	*n*	Mean (SD)	*n*	Mean (SD)	*n*	Mean (SD)	*n*	Mean (SD)
Psychological	4.52 (5.38)	360	3.28 (4.49)	320	5.12 (5.48)	80	7.67 (5.95)				
Physical	1.00 (3.41)	280	0.38 (2.15)	400	1.13 (3.31)	80	2.17 (4.29)				
Sexual	0.73 (1.65)	120	0.09 (0.45)	280	0.31 (1.03)	120	0.55 (1.64)	200	1.49 (2.09)	40	2.20 (2.30)
Economic	0.23 (0.77)	440	0.07 (0.42)	160	0.25 (0.88)	160	0.63 (1.13)				
Total	6.43 (8.74)	280	3.60 (6.00)	400	7.23 (9.15)	80	12.35 (10.80)				

SD, standard deviation; IPV, intimate partner violence; CUHSCs, comprehensive urban health service centers.

In Table 6, the demographic and social variables are compared in three subgroups resulting from the total violence score. As can be seen, the three subgroups are significantly different in terms of the woman’s age and education, husband’s education and occupation, housing status and monthly household income. In subgroups 3 and 2, which has a higher level of violence, the education level of a smaller number of women and their spouses are university-level, and the percentage of uneducated women or spouses was higher. The level of violence was higher in men with self-employment or workers and with a monthly household income level of less than 5 million Tomans. Also, the age of women in the subgroup with violence was lower.

**Table 6:** Compare of demographic and social variables in three subgroups resulting from of the total IPV score.

Variable	Category	Subgroup 1 (*n* = 280)	Subgroup 2 (*n* = 400)	Subgroup 3 (*n* = 80)	Test statistic	*p*-value
**Women**						
Age	–	37.22 (8.52)	34.48 (8.80)	34.58 (8.39)	19.43	<0.001^a^
Education level	Illiterate	12 (4.3)	32 (8.0)	12 (15.0)	44.30	<0.001^b^
	Under diploma	75 (26.8)	137 (34.3)	18 (22.5)		
	Diploma	78 (27.9)	133 (33.3)	36 (45.0)		
	Bachelor	88 (31.4)	82 (20.5)	8 (10.0)		
	Master/PhD	27 (9.6)	16 (4.0)	6 (7.5)		
Job status	Housewife	225 (80.4)	343 (85.6)	72 (90.0)	5.86	0.053^b^
	Employed	55 (19.6)	57 (14.2)	8 (10.0)		
Addiction status	Yes	1 (0.4)	0	0	–	–
	No	279 (99.6)	400 (100)	80 (100)		
**Husband**						
Education level	Illiterate	11 (3.9)	29 (7.2)	17 (21.3)	45.79	<0.001^b^
	Under diploma	71 (25.4)	135 (33.8)	25 (31.3)		
	Diploma	85 (30.4)	126 (31.5)	23 (28.7)		
	Bachelor	79 (28.2)	83 (20.8)	7 (8.8)		
	Master/PhD	34 (12.1)	27 (6.8)	8 (10.0)		
Job status	Unemployed	1 (0.4)	9 (2.3)	2 (2.5)	14.57	0.026^c^
	Government job	97 (34.6)	119 (29.8)	15 (18.8)		
	Self-employed	171 (61.1)	245 (61.3)	60 (75.0)		
	Daily worker	11 (3.9)	27 (6.8)	3 (3.8)		
Addiction status	Yes	3 (1.1)	8 (2.0)	1 (1.3)	0.98	0.660^c^
	No	277 (98.9)	392 (98.0)	79 (98.8)		
**Household**						
Housing ownership status	Rental	82 (29.3)	167 (41.8)	42 (52.5)	18.47	<0.001^b^
	Owner	198 (70.7)	233 (58.3)	38 (47.5)		
Monthly income (million Tomans)	< 5	23 (8.2)	101 (25.3)	56 (70.0)	172.67	<0.001^b^
	5–10	183 (65.4)	272 (68.0)	20 (25.0)		
	10–15	35 (12.5)	13 (3.3)	2 (2.5)		
	> 15	39 (13.9)	14 (3.5)	2 (2.5)		

a Reported as mean (SD, standard deviation) with Kruskal-Wallis test.

b Reported as frequency (%) with Chi-square test.

c Reported as frequency (%) with Chi-square test based on Monte Carlo Simulation.

## Discussion

This study was conducted with the aim of investigating the IPV prevalence in Zanjan city based on CUHSCs. We found that about 80% of the participants had experienced at least one type of IPV in the past year. Meanwhile, only 5% had experienced all four types of violence. Due to the importance of this issue and the impact that DV against women has on women’s health, various studies have been conducted around the world to investigate the current situation and the factors that affecting it. Sardinha et al., in a systematic review, reported that 27% of women aged 15–49 have experienced physical or sexual violence or both in their lifetime. Also, 13% experienced both of these types of violence in the past year (Sardinha et al., 2022). The DV rate against women in the Middle East region, where Iran is located, is reported to be 31% in their lifetime and 16% in the past year. The prevalence of physical and/or sexual violence in the past year in Iran was 15–19% (Sardinha et al., 2022). Our study shows that last year’s IPV rate was nearly 80% and six, five, four times higher than global data, in Middle East region, and in Iran, respectively. The reason for the difference between these data may be due to the time period of our survey that was conducted in 2021. The 2020 coronavirus disease (COVID-19) pandemic, during which limited and extended lockdowns at times, may justify high IPV rates in this study. For example, the 51^st^ issue of Weekly Epidemiological Update on COVID-19 points out that the deaths of people infected with COVID-19 in Iran have increased by 34%, which led to the imposition of activity restrictions (World Health Organization, 2021). Various studies have shown that IPV increases during crises such as financial, environmental and socio-political crises. The COVID-19 pandemic has had a significant impact on living with DV, often exacerbating the violence experiences (Lyons and Brewer, 2022). Quarantine and isolation of the victim with her abuser exposes her to a special risk (Sutton and Beech, 2023). Another reason for the difference in prevalence is that we investigated four types of DV against women, while other studies mostly investigated physical or sexual violence or both. The prevalence of this health problem has been reported differently in different geographical areas. For example, in Tanzania, 26.5% experienced physical and sexual violence, and in Pakistan, 88.8% of the participants mentioned physical, psychological and sexual violence (Kapiga et al., 2017; Hussain et al., 2020). 15.6% of women aged 16 and older living in Spain experienced psychological, physical or sexual violence before the COVID-19 pandemic (Sanz-Barbero et al., 2019).

According to our research, the types of IPV against women in Zanjan city are: psychological (highest), sexual, physical and economic (lowest). Most of the psychological violence is mild violence, indicating that at least one answer is positive in 1–5 acts of the questionnaire. Similar to the results of the present study, psychological violence is the most common type of violence against women compared to physical and sexual violence in Europe, America and Western societies (Martín-Fernández et al., 2019; Dokkedahl et al., 2022). Psychological violence usually precedes physical violence and its prevalence is expressed differently in different societies, depending on how it is defined and measured in different societies and cultures. In Europe, this value is given 10–90% (Martín-Fernández et al., 2019).

In the current study, IPV was conducted based on 19 CUHSCs. It was observed that CUHSCs 1, 2, 15, 16 have the highest overall violence, and CUHSCs 14 had the lowest overall violence. CUHSCs 1 and 2 are more densely populated than CUHSC 14, are located in the southern part of the city, and CUHSCs 1, 2, 15, 16 have located in low socio-economic areas. Meanwhile, the 14th CUHSC is located in the high socio-economic area in the northern part of the city and distinct cultural and socio-economic differences compared to these centers. Based on the analysis of CUHSCs and its division into homogeneous subgroups, subgroup 3 has the highest violence level and the average age of women is lower than subgroup 1. These CUHSCs have lower university education of women and their spouses, women employment, monthly household income and lower-level education of women and their spouses, private house, and higher unemployment rates of women’ spouses.

The differences of IPV in different geographical regions can be attributed to socio-economic and cultural differences in different regions. In a study conducted in Pakistan that prevalence of intimate partner violence, poverty has the most impact on violence against women. Then, factors such as influence of in-law, second marriage, stepchildren, forced intimate relationships, husband’s irresponsibility, addiction and having a disabled child have been effective (Hussain et al., 2020). As mentioned earlier, in our results, the level of education of women and their spouses, monthly income and women’s employment status were important variables for determining the socio-economic status of women. The household income level of women living in the sub-group who were subjected to violence was low, and no statistically significant effect was observed regarding the addiction due to its low prevalence in the individual and his wife.

Women who suffered sexual or physical abuse in childhood will also experience physical, sexual and psychological violence from their husbands in adulthood, and this factor increases the risk of IPV 3–4 times (Sanz-Barbero et al., 2019). Inequalities of people’s resources and income both in the family and at the community level cause more abuse (Cools and Kotsadam, 2017). DV against women is more prevalent in areas with low to middle income than in areas with high income levels. In areas with low socio-economic status, due to the economic insecurity that women experience, as well as cultural reasons and social stigma and insufficient support services to support women who are subjected to violence, people in stay in a relationship that is accompanied by violence (Sardinha et al., 2022). There is evidence of a non-linear, U-shaped relationship between women’s education level and the risk of violence. Higher levels of education are associated with lower rates of perpetrating violence and being a victim of IPV. Also, women with less educated have a lower risk of violence compared to women with more education at the secondary and pre-university levels. This is probably because those who study less are less likely to challenge their partners and ultimately will be less violent (Fulu and Heise, 2015).

Many factors have been mentioned as risk factors of DV against women in various studies. According to a systematic review study conducted in 2023, these factors have been mentioned in three groups. Individual factors such as age, level of education, consumption of alcohol and drugs, and history of violence in family in the victim and the violent person. Factors related to the relationship such as the level of gender inequality in the relationship with the spouse and suspicion of infidelity can also cause DV against women. In a relationship where there is gender equality, a person is less likely to be abused. Other influencing factors include household-level and community-level factors. Having at least one child increases the risk. Village living and lack of social support are also risk factors for DV against women (Brown et al., 2023; Ghoshal et al., 2023).

Given that many health consequences of a people exposed to DV (Bacchus et al., 2018), comprehensive and urgent interventions are needed in high-risk areas. In our study, about 5% of participants mentioned severe psychological and physical violence requiring precautions against possible violent injury. Regarding sexual violence, 14.5% of the women in the study were victims of serious sexual violence. Interventions should be done in different areas. Women’s empowerment interventions can help increase women’s self-efficacy and focus on improving skills and connecting with social support centers. Financial interventions aimed at helping them undertake income-generating activities can make them financially independent (Babaee et al., 2021). Women’s empowerment and gender equality is the fifth Sustainable Development Goal that must be achieved by 2030. Under this document, countries commit to reducing all forms of violence against women in public and private context (Johnston, 2016).

Due to the complex nature of intimate partner violence and attention to the fact that domestic violence against women is not only an expression of men’s power and superiority over women, but also a result of social laws that cause men to dominate women, intervention and preventive strategies are carried out at different levels of society, community, interpersonal relationships and at the individual level (Michau et al., 2015; Sangeetha et al., 2022). Interventions at the society level emphasize on supporting the change of discriminatory laws and ensuring that laws and policies support women who are subjected to violence. At the community level, healthcare providers are one of the first people outside the family who can see the symptoms of violence. Studies have shown that usually in the early stages of violence, it is more likely to refer to the healthcare system. Healthcare providers can identify her, and it is important to educate these people on how to respond, and educational interventions to increase the awareness and attitudes and practices of healthcare providers are important and can improve health outcomes in women who have been subjected to violence (Michau et al., 2015; Kalra et al., 2021).

The goal community-level interventions are to create an equal and violence-free environment for women. Because the presence and response to intimate partner violence depends on social norms regarding power and gender (such as male authority, acceptance of wife beating, and female obedience), and these norms can support or condemn violence. The role of the man as the provider of the woman, sexual activity as a sign of masculinity and the shamefulness of divorce are effective (Michau et al., 2015). In our study, it was also observed that sexual violence is the second type of violence that is probably due to the mentioned reasons and requires intervention in the field of changing the attitude of the IPV Offenders.

Another intervention strategy is programs to change and improve interpersonal communication. Individual attitudes and behaviors are formed in the family, and planning at this level should be implemented with the aim of supporting men and boys to encourage more equitable gender power relations and support the leadership and participation of women and girls (Michau et al., 2015). At the individual level, individual behaviors and attitudes, such as adherence to traditional male and female norms and indifference to violence and fear of intervention by women, contribute to the continuation of interpersonal violence, and aspirational programming can help these people to imagine a positive and fair perspective in relationships (Michau et al., 2015). Exposure to violence in childhood can also be one of the factors that can cause violence in the future. Boys who were punished in childhood or witnessed their mother being beaten have a high probability of violence against women, and programs should be made to prevent child abuse and such anomalies in the family (Heise, 2011). Intimate Partner Violence Offenders should also participate in intervention programs to prevent future violence. These people often have low motivation to change and deny their violent behavior because they have usually participated in intervention programs by introducing legal authorities and not voluntarily. It has been observed in the systematic review that interventions with motivational strategies including stage of change-based treatment, strengths-based treatment, motivational interviewing and retention techniques can increase the effectiveness of interventions. The results of this study have shown that interventions without motivational strategies can increase the probability of dropout these interventions by 1.73 times (Santirso et al., 2020).

## Conclusion

According to the results, only 20% of women have not experienced any type of violence and the most common type of violence was psychological violence. CUHSCs with a low socio-economic level had a higher total violence than highest socio-economic level.

Finally, health policy makers should design and implement intervention measures based on high-risk areas and Social Determinant of Health. The implementation of first-level preventive interventions and educational classes can help to reduce the incidence of DV against women. Also, second-level preventive interventions and early screening and identification of people exposed to IPV can prevent possible injuries. Also, designing and conducting interventional studies and research on the impact of these interventions in community will be helpful in future studies.

## Data availability statement

The original contributions presented in the study are included in the article/supplementary material, further inquiries can be directed to the corresponding author/s.

## Ethics statement

The studies involving humans were approved by Zanjan University of Medical Sciences with ethics code IR.ZUMS.REC.1400.474. The studies were conducted in accordance with the local legislation and institutional requirements. The participants provided their written informed consent to participate in this study.

## Author contributions

FK: Conceptualization, Investigation, Methodology, Project administration, Supervision, Writing – original draft, Writing – review & editing. FA: Data curation, Formal analysis, Methodology, Project administration, Software, Visualization, Writing – original draft, Writing – review & editing. VFA: Data curation, Formal analysis, Software, Visualization, Writing – original draft, Writing – review & editing.
